# Rapid Nipah virus entry into the central nervous system of hamsters via the olfactory route

**DOI:** 10.1038/srep00736

**Published:** 2012-10-15

**Authors:** Vincent J. Munster, Joseph B. Prescott, Trenton Bushmaker, Dan Long, Rebecca Rosenke, Tina Thomas, Dana Scott, Elizabeth R. Fischer, Heinz Feldmann, Emmie de Wit

**Affiliations:** 1 Laboratory of Virology, Microscopy Unit, Division of Intramural Research, National Institute of Allergy and Infectious Diseases, National Institutes of Health, Hamilton, MT, USA; 2 Rocky Mountain Veterinary Branch, Microscopy Unit, Division of Intramural Research, National Institute of Allergy and Infectious Diseases, National Institutes of Health, Hamilton, MT, USA; 3 Research Technologies Branch, Microscopy Unit, Division of Intramural Research, National Institute of Allergy and Infectious Diseases, National Institutes of Health, Hamilton, MT, USA; 4 Department of Medical Microbiology, University of Manitoba, Winnipeg, Manitoba, Canada

## Abstract

Encephalitis is a hallmark of Nipah virus (NiV) infection in humans. The exact route of entry of NiV into the central nervous system (CNS) is unknown. Here, we performed a spatio-temporal analysis of NiV entry into the CNS of hamsters. NiV initially predominantly targeted the olfactory epithelium in the nasal turbinates. From there, NiV infected neurons were visible extending through the cribriform plate into the olfactory bulb, providing direct evidence of rapid CNS entry. Subsequently, NiV disseminated to the olfactory tubercle and throughout the ventral cortex. Transmission electron microscopy on brain tissue showed extravasation of plasma cells, neuronal degeneration and nucleocapsid inclusions in affected tissue and axons, providing further evidence for axonal transport of NiV. NiV entry into the CNS coincided with the occurrence of respiratory disease, suggesting that the initial entry of NiV into the CNS occurs simultaneously with, rather than as a result of, systemic virus replication.

Nipah virus is a member of the *Henipavirus* genus in the family *Paramyxoviridae*. Like several other paramyxoviruses, Nipah virus causes encephalitis in infected individuals. During the first documented Nipah virus outbreak in Malaysia and Singapore in 1998–1999, 276 cases of Nipah virus encephalitis were observed, with 106 fatalities^1^. In subsequent Nipah virus outbreaks in Bangladesh, a larger proportion of respiratory disease was observed than in Malaysia, but neurological symptoms were also common^2^,^3^. Follow-up studies have shown that approximately 19% of patients that survived Nipah virus infection still suffered long-term neurological deficits that lasted more than four months after the initial outbreak^4^,^5^. Moreover, a small proportion of patients experienced relapse or late-onset encephalitis that occurred up to many years after the initial infection from which they had recovered^6^,^7^,^8^. Autopsies have shown vasculitis of the small arteries, arterioles, capillaries and venules of the central nervous system (CNS) in patients with Nipah virus encephalitis^9^. Presence of viral inclusions was another common observation in the CNS of deceased patients during the Malaysian outbreak^9^.

Although it is evident that Nipah virus can replicate in the CNS, it is currently unknown how Nipah virus reaches the cells of the central nervous system. Canine distemper virus, a member of the closely related genus *Morbillivirus* in the family *Paramyxoviridae*, has been shown to travel to the CNS via anterograde transport along olfactory neurons in the nasal cavity, and via the hematogenous pathway, probably through infected lymphocytes^10^. Recently, it was proposed that Hendra virus enters the brain via olfactory neurons in an aged mouse model of Hendra virus encephalitis^11^.

Recently, it has been described that Nipah virus can disseminate via binding to leukocytes; however, the route of entry of virus into the CNS was not specifically addressed in this study^12^. A study of experimental Nipah virus infection of pigs suggested that Nipah virus reaches the CNS through cranial nerves and via hematogenous spread^13^. Here, we followed the progression of Nipah virus upon intranasal inoculation from the nasal epithelium into the CNS in the Syrian hamster model and showed that transport of virus along neurons in the olfactory epithelium into the brain is one pathway used by Nipah virus to enter the CNS and cause encephalitis. Entry of Nipah virus into the CNS occurred rapidly, within 4 days of inoculation.

## Results

### Nipah virus infection of hamsters

In a previous study we determined that intranasal inoculation of hamsters with 10^5^ TCID_50_ of Nipah virus, strain Malaysia resulted in an initial respiratory phase of infection after which a proportion of surviving hamsters developed neurological signs of disease^14^. Therefore, we inoculated 32 hamsters intranasally with 10^5^ TCID_50_ of Nipah virus. On 2, 4, 6, 8, 10 and 12 days post inoculation (dpi) four hamsters each were euthanized and necropsied to look at the progression of Nipah virus infection. Six hamsters developed obvious neurological signs such as paralysis, seizures, torticollis and tremors between 7 and 12 dpi and were euthanized according to approved protocol.

### Progress of Nipah virus infection in the nasal turbinates

**Figure 1:**
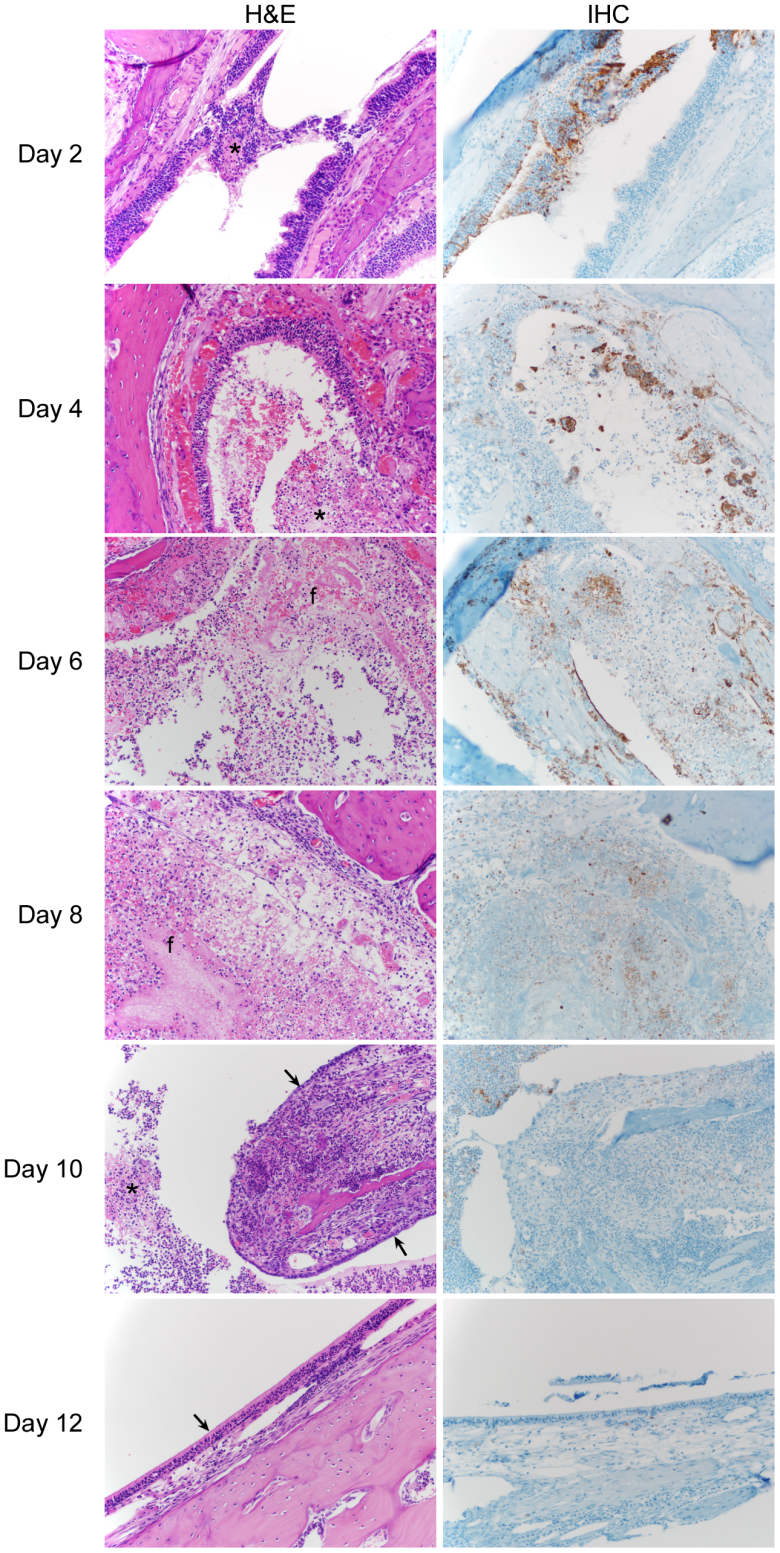
Histopathological and immunohistochemical analysis of the progress of Nipah virus infection of the olfactory epithelium of hamsters. Hamsters were inoculated intranasally with 10^5^ TCID_50_ of Nipah virus and tissue samples were collected at 2, 4, 6, 8, 10 and 12 days post inoculation. Tissue sections were stained with hematoxylin-eosin (H&E, left panels) or a monoclonal antibody against Nipah virus nucleoprotein (IHC, right panels), which is visible as a red-brown staining. In the H&E panels, asterisks indicate inflammation and necrosis; ‘f’ depicts areas with fibrin deposits and arrows indicate early (day 10) and advanced (day 12) regeneration of epithelium. H&E and IHC sections were from sequential sagittal sections. A schematic representation of olfactory and central nervous system architecture is shown in figure 7; uninfected control tissue can be found in Supplementary Fig. S1 online.

Beginning at 2 dpi, the nasal turbinates of every hamster in the study demonstrated inflammation and necrosis of either the respiratory epithelium, olfactory epithelium or both. The lesions were most severe in the olfactory epithelium (Fig. 1 and Table S1) and increased in severity over time, with the most severe lesions present between 8 and 10 dpi. On 12 dpi the epithelium had largely regenerated and was associated with significantly less inflammation. The epithelial lesions were characterized by loss of epithelial cells resulting in ulcers that were covered by cellular and karyorrhectic debris, degenerate neutrophils and macrophages, mats of fibrin and haemorrhage. There were numerous syncytial cells within the adjacent epithelium. Multifocally, submucosal glands were replaced by similar necrotic debris and inflammation; syncytial cells could also be found. Immunohistochemistry revealed abundant viral antigen distributed throughout these lesions; however, the majority of the antigen was present in olfactory epithelium (Fig. 1 and Table S2).

### Entry of Nipah virus into the CNS

**Figure 2:**
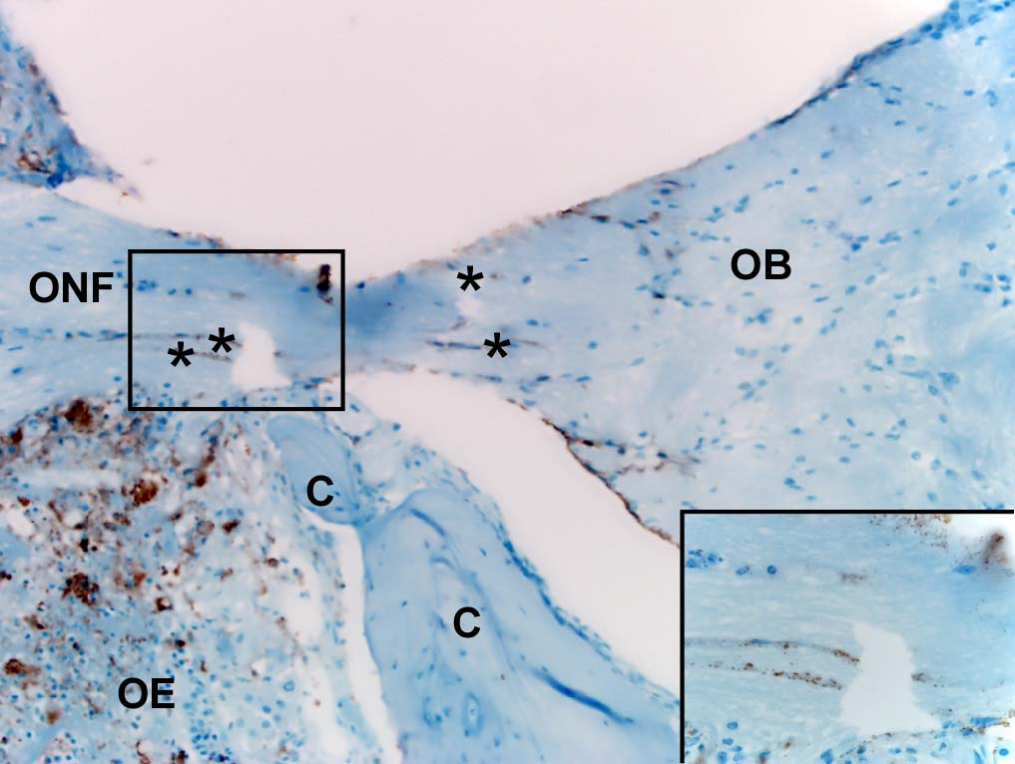
Nipah virus-infected neurons extending through the cribriform plate. Tissues were collected on 4 dpi and stained with a monoclonal antibody against Nipah virus nucleoprotein, which is visible as a red-brown staining. Asterisks indicate positive neurons within the olfactory nerve fibre (ONF), crossing from the olfactory epithelium (OE) to the olfactory bulb (OB) through the cribriform plate (C). The inset shows a higher magnification of the boxed area with antigen-positive neurons. A schematic representation of olfactory and central nervous system architecture is shown in figure 7.

Shortly after detection of Nipah virus in the nasal turbinates, the virus entered the CNS. Single Nipah virus infected neurons extending from the olfactory epithelium through the cribriform plate into the olfactory bulb were first detected at 4 dpi, providing direct evidence for transport of Nipah virus along the olfactory neurons into the CNS (Fig. 2).

**Figure 3:**
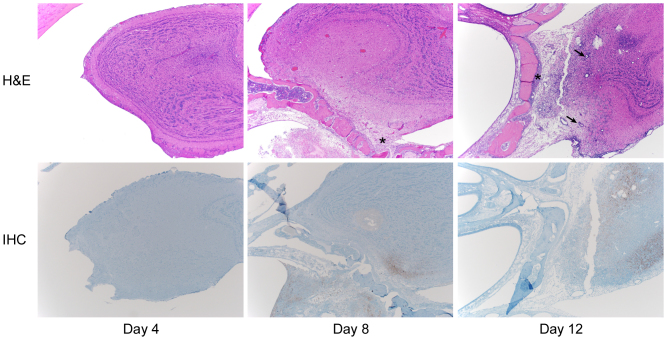
Histopathological and immunohistochemical analysis of the progress of Nipah virus infection of the olfactory bulb of hamsters. Hamsters were inoculated intranasally with 10^5^ TCID_50_ of Nipah virus and tissue samples were collected at 4, 8 and 12 days post inoculation. Tissue sections were stained with hematoxylin-eosin (H&E, top panels) or a monoclonal antibody against Nipah virus nucleoprotein (IHC, bottom panels), which is visible as a red-brown staining. The asterisks in the H&E panels indicate inflammation and edema; arrows indicate perivascular cuffing. H&E and IHC sections were from sequential sagittal sections. A schematic representation of olfactory and central nervous system architecture is shown in figure 7; uninfected control tissue can be found in Supplementary Fig. S1 online.

On 6 dpi the olfactory bulbs demonstrated a mild meningoencephalitis that increased in severity over time (Fig. 3 and Table S1). The meningoencephalitis was characterized by multifocal meningeal and perivascular infiltrates of lymphocytes, with scattered necrotic neurons mixed with small numbers of degenerate neutrophils, microglia and gitter cells. There were multifocal glial nodules in affected areas. Immunohistochemistry revealed abundant viral antigen in the olfactory bulbs by 8 dpi which was most abundant in the area immediately adjacent to the cribriform plate (Fig. 3 and Table S2). In the olfactory bulb, neurons and axons of the olfactory nerve layer, glomerular layer and external plexiform layer were Nipah virus antigen positive. Less often neurons and axons from the internal plexiform layer and granule cell layer were affected. By 12 dpi viral antigen was seen throughout the olfactory bulb.

### Dissemination of Nipah virus throughout the CNS

**Figure 4:**
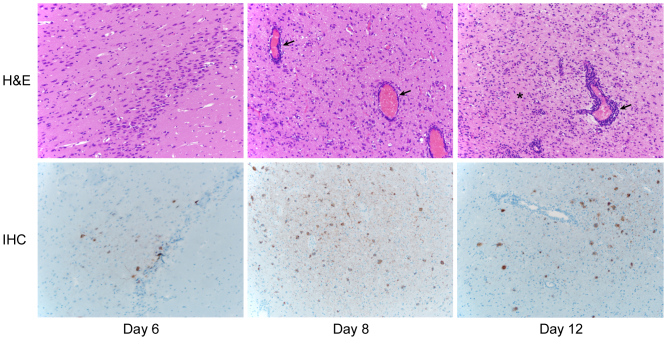
Histopathological and immunohistochemical analysis of the progress of Nipah virus infection of the CNS of hamsters. Hamsters were inoculated intranasally with 10^5^ TCID_50_ of Nipah virus and tissue samples were collected at 6, 8 and 12 days post inoculation. Tissue sections of the ventral cortex were stained with hematoxylin-eosin (H&E, top panels) or a monoclonal antibody against Nipah virus nucleoprotein (IHC, bottom panels), which is visible as a red-brown staining. The asterisks in the H&E panels indicate gliosis with infiltrated astrocytes, glial cells and microglia; arrows indicate perivascular cuffing. H&E and IHC sections were from sequential sagittal sections. A schematic representation of olfactory and central nervous system architecture is shown in figure 7; uninfected control tissue can be found in Supplementary Fig. S1 online.

**Figure 5:**
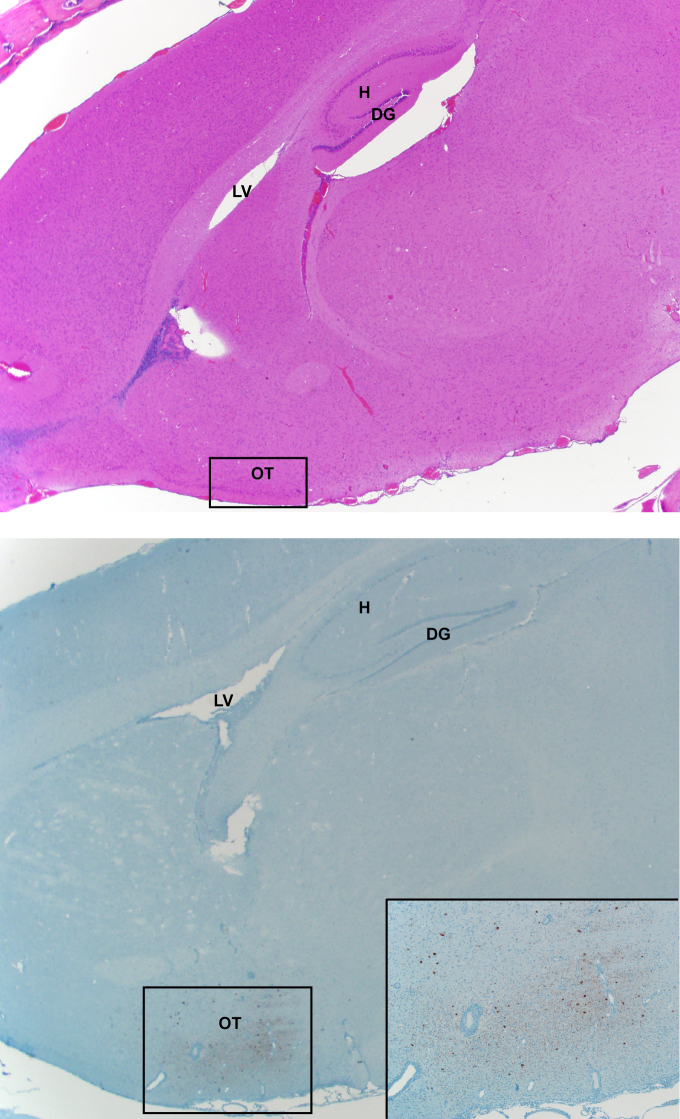
Nipah virus infection of neurons of the olfactory tubercle. Tissues were collected on 12 dpi and stained with hematoxylin-eosin (top panel) or a monoclonal antibody against Nipah virus nucleoprotein, which is visible as a red-brown staining (bottom panel).The inset in the bottom panel shows a close-up view of the area of the olfactory tubercle (OT). LV: lateral ventricle; H: hippocampus; DG: dentate gyrus. H&E and IHC sections were from sequential sagittal sections. A schematic representation of olfactory and central nervous system architecture is shown in figure 7.

Nipah virus dissemination throughout the CNS started as early as 6 dpi; in addition to olfactory bulb inflammation there were multifocal areas of encephalitis (Fig. 4 and Table S1). Histologically these lesions resembled those described in the olfactory bulb and were centred in the area of the olfactory tubercle, which is directly connected to the olfactory bulb. Immunohistochemical analysis demonstrated viral antigen in neurons within affected areas of the olfactory tubercle (Fig. 5 and Table S2). From the olfactory tubercle Nipah virus spread further into the ventral cortex and was eventually detected in the ventral cerebrum and immediately adjacent to the lateral ventricles. Cerebral vasculitis with fibrin thrombi and haemorrhage was noted in one animal; otherwise, brain lesions were limited to perivascular infiltrates of lymphocytes and plasma cells, with scattered necrotic neurons, gliosis, and neuronal degeneration. In the CNS, only neurons were immunopositive; there was no Nipah virus antigen present in cells surrounding affected vessels.

### Ultrastructural changes in the CNS of Nipah virus infected hamsters

**Figure 6:**
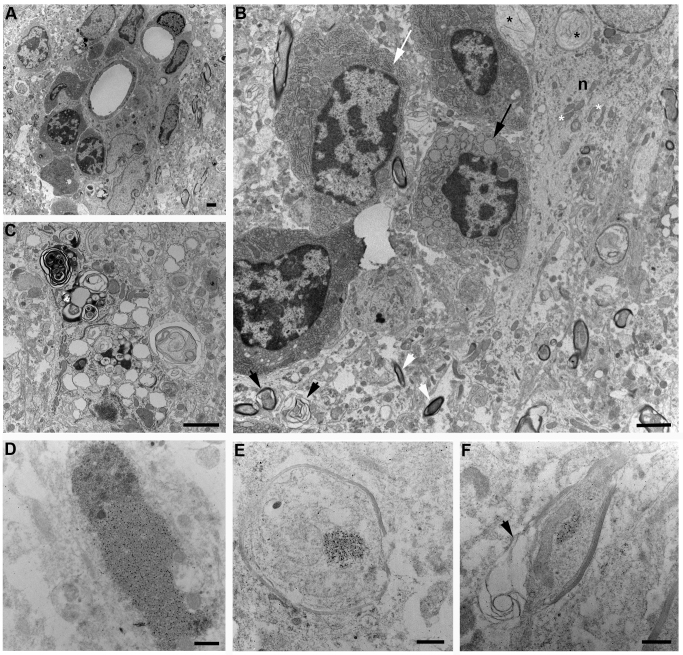
Ultrastructural analysis of Nipah virus replication in the hamster brain. Samples of the brain of Nipah virus infected hamsters were collected at 12 days post inoculation. Examination of the ventral cortex revealed perivascular inflammation with extravasation of plasma cells and monocyte (A); degeneration of neurons (n) with mitochondrial swelling (black asterisk), dilation of rough endoplasmic reticulum (RER; black arrow) in an adjacent plasma cell and axonal myelin degradation (black arrowhead) (B); phospholipid whorls and vacuolization in a neuron (C). Immune labelling of tissue sections using a monoclonal antibody against Nipah virus nucleoprotein revealed aggregations of nucleocapsids (D). Nucleoprotein aggregates were also observed in axons (E, F), some of which showed myelin degradation (F). For comparison, ‘normal’ mitochondria (white asterisk) and RER (white arrow) are indicated; white arrowheads indicate axons showing no pathology in this plane of sectioning. Scale bars in (A), (B) and (C) represent 2µm, bars in (D), (E) and (F) represent 0.5 µm.

Cellular pathology of the CNS was investigated by transmission electron microscopy (TEM) examination of the ventral cortex in the area of the olfactory tubercle. Examination of these tissues collected on 12 dpi revealed several ultrastructural pathological changes. Marked extravasation of plasma cells from blood vessels was observed (Fig. 6A), indicating active recruitment of immune cells to the brain. In some cases plasma cells in areas showing pathology displayed signs of degeneration such as dilated rough endoplasmic reticulum (Fig. 6B). Mitochondrial swelling, vacuolization and phospholipid whorls were observed in neurons; axons showed different stages of myelin degradation (Fig. 6B and 6C). In areas of tissue damage we observed aggregates of electron-dense material resembling Nipah virus nucleoprotein inclusions as described by others^9^,^15^,^16^,^17^. These aggregates were also seen in axons and often consisted of an electron-dense area flanked by less-dense material. Through immune labelling using an α-N antibody we confirmed that the aggregates of electron-dense material were nucleoprotein inclusions (Fig. 6D–F). No mature virus particles were observed.

## Discussion

**Figure 7:**
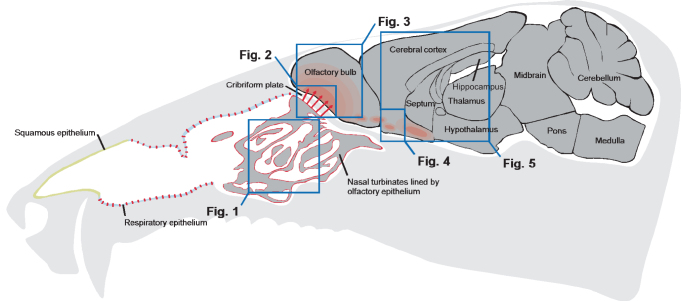
Schematic representation of Nipah virus entry into the CNS via the olfactory route. Nipah virus infection is initiated in the respiratory and olfactory epithelium in the nasal turbinates following intranasal inoculation. As early as 4 dpi Nipah virus enters the CNS via neurons extending from the olfactory epithelium through the cribriform plate into the olfactory bulb. The olfactory bulb immediately adjacent to the olfactory epithelium becomes infected between 4 and 8 dpi, and from there the virus spreads throughout the remainder of the olfactory bulb. Following infection of the olfactory bulb, the olfactory tubercle region is the first area of the CNS to become infected as early as 6 dpi. The locations of Nipah virus replication as determined by immunohistochemistry are indicated in red. The boxed areas represent the spatial orientation of the histopathology depicted in figures 1 through 5.

In this study, we clearly showed the rapid progression of Nipah virus from the olfactory epithelium in the nasal turbinates to the CNS following intranasal inoculation, as represented schematically in Figure 7. As early as 4 dpi neurons extending from the olfactory epithelium through the cribriform plate into the olfactory bulb, were shown to be infected with Nipah virus. Temporal analysis showed that the area of the olfactory bulb immediately adjacent to the olfactory epithelium became infected between 4 and 8 dpi, and from there the virus spread throughout the remainder of the olfactory bulb. Axons forming the lateral olfactory tract and tufted cells from the ventral portion of the olfactory bulb project from the olfactory bulb into the olfactory tubercle^18^. It is therefore not surprising that the olfactory tubercle region was the first area of the CNS to become infected as early as 6 dpi, following infection of the olfactory bulb. The olfactory tubercle is interconnected with the limbic system and thereby offers many opportunities for Nipah virus to spread further throughout the CNS. For example, in a previous study using the same virus, dose and route of inoculation Nipah virus extended into the pons in an animal displaying neurological signs (data not shown).

Based on the observed vasculitis in the CNS of deceased patients, it was proposed that Nipah virus accesses the CNS through the hematogenous route^9^; replication in cerebrovascular endothelial cells could result in disruption of the blood-brain barrier and entry of Nipah virus into the CNS. These observations were based on fatal cases of Nipah virus encephalitis and these observations in the late stage of disease do not necessarily reflect early stages in disease development such as the initial entry of Nipah virus into the CNS. In the hamster model described here, vasculitis was rarely observed in the CNS and only in late stages of disease. Although syncytial endothelial cells were common in the lungs of Nipah virus infected hamsters (data not shown), they were not detected in the central nervous system.

Disruption of the blood-brain barrier has been shown to occur in hamsters infected with Nipah virus, but only in animals with neurological signs of disease^19^, implicating that disruption of the blood-brain barrier was not the initial point of entry of Nipah virus into the CNS. Rather, the disruption of the blood-brain barrier could be the result of virus replication in the CNS. It has been shown that cytokines, such as IL-6, TNF-α, IL-1α, IL-1β and IFN-γ produced during virus replication in the CNS, can result in breakdown of the blood-brain barrier (reviewed in^20^). In agreement with this, TNF-α and IL-1β expression were shown to be upregulated in the brains of Nipah virus infected hamsters^19^. The study described here does not address whether the olfactory route is the only entry route used by Nipah virus in the hamster model. In contrast, the olfactory bulbs of nine deceased patients did not show histopathology^9^ implying that the olfactory route might not have been the route of Nipah virus entry into the brain in these cases. This is partly supported by the recently established African green monkey model of Nipah virus infection using intratracheal inoculation^21^. Consistent detection of viral RNA in the plasma of the animals suggested the potential for the hematogenous route for CNS entry^21^. However, the detection of Nipah virus antigen and associated pathology in the upper respiratory tract tissues of these animals indicates the ability of Nipah virus to enter the upper respiratory tract upon inoculation of the lower respiratory tract^21^. This is supported by the detection of viral RNA in nose and throat swabs collected from the animals, suggesting that even upon intratracheal inoculation the virus can reach the nasal cavity and could subsequently enter the CNS via the olfactory route.

The recent finding that leukocytes can transport Nipah virus without becoming infected^12^ in combination with findings that the expression of CXCL10, a chemoattractant for leukocytes, is upregulated in the CNS of humans and hamsters infected with Nipah virus^19^,^22^ is a potential second route of entry of Nipah virus into the CNS that should be investigated. Experimental infections in pigs indeed suggested entry of Nipah virus into the CNS via cranial nerves, including the olfactory nerve, and by crossing the blood-brain barrier^13^.

It is currently unclear whether CNS entry routes observed in Nipah virus animal models with a relatively large area of olfactory epithelium, such as pigs, ferrets and the Syrian hamster, can be extrapolated to Nipah virus entry into the human CNS. The increased surface area of the olfactory epithelium of these animal models may result in a higher incidence of CNS infection via the olfactory route. However, this does not exclude that the olfactory route could be used for Nipah virus entry into the human CNS. The involvement of the olfactory pathway in CNS entry by other human viruses (e.g. human herpesvirus-6, Borna disease virus and rabies virus) indicates that certain viruses can efficiently use this route to enter the human CNS^23^,^24^. These observations in humans are generally well reproduced in animal models with a relatively large area of olfactory epithelium, such as mice and rats. The recently established African green monkey model^21^, with a relatively small olfactory epithelium, uses the intratracheal route of inoculation, hampering direct comparison between animal models with different olfactory epithelium surface areas. However, the African green monkey model would be suitable to address the potential predisposition to the CNS entry via the olfactory route in animal models with a relatively large area of olfactory epithelium vs. animal models with a relatively small area of olfactory epithelium.

Whereas it is not clear how differences in architecture and physiology of the olfactory system affect Nipah virus CNS entry via the olfactory route, it seems logical that the intranasal inoculation used in this study does predispose the animals to efficient replication in the upper respiratory epithelium and the olfactory epithelium as opposed to either intratracheal or intraperitoneal inoculation routes used in other studies. This could result in a bias towards CNS entry via the olfactory route. However, the initiation of Nipah virus nasal shedding before oropharyngeal shedding upon natural infection via contact transmission^14^ suggests that the intranasal route of inoculation is the best approximation of a natural route of infection and underscores the potential of Nipah virus to gain access to the upper respiratory tract.

By EM, we clearly observed nucleoprotein aggregates in axons in the brain of infected animals, again suggesting that Nipah virus utilizes axonal transport for rapid entry of, and dissemination through, the CNS. Although many nucleoprotein aggregates were observed, no mature virus particles could be seen. This observation is in line with several studies on brain material of patients from the Nipah virus outbreak in Malaysia that also described nucleoprotein inclusions but no virus particles^9^,^16^,^17^; however, infectious virus has been isolated from hamsters inoculated intranasally with Nipah virus in the past^19^.

The rapid appearance of Nipah virus in the CNS coincided with the first signs of bronchointerstitial pneumonia between 2 and 4 dpi (Table S1 and S2), suggesting that the initial entry of Nipah virus into the CNS occurs during the respiratory stage of disease. The epithelium of the upper respiratory tract likely functioned as the initial porte d'entrée for Nipah virus and virus was subsequently disseminated to the CNS via the olfactory route and to the lower respiratory tract via the trachea. The rapid entry of Nipah virus into the CNS emphasizes the need for treatment focussed not only at alleviating the respiratory phase of disease but that is also effective in the CNS to prevent the later onset of neurological disease.

## Methods

### Ethics statement

All animal experiments were approved by the Institutional Animal Care and Use Committee of the Rocky Mountain Laboratories, and performed following the guidelines of the Association for Assessment and Accreditation of Laboratory Animal Care, International (AAALAC) by certified staff in an AAALAC-approved facility.

### Virus and cells

Nipah virus (strain Malaysia) was kindly provided by the Special Pathogens Branch of the enters for Disease Control and Prevention, Atlanta, Georgia, United States and propagated in VeroE6 cells in DMEM (Sigma) supplemented with 10% fetal calf serum (Hyclone, Logan), 1 mM L-glutamine (Lonza), 50 U/ml penicillin and 50 μg/ml streptomycin (Gibco).

### Animal experiments

Eight groups of four 6-8 week-old female Syrian hamsters (HsdHan^tm^:AURA, Harlan Laboratories) were inoculated intranasally with 10^5^ TCID_50_ of Nipah virus in a total volume of 80 µl. On days 2, 4, 6, 8, 10 and 12, one group of hamsters was euthanized. Remaining hamsters were euthanized upon displaying neurological signs of disease and processed for histopathology or electron microscopic analysis. Hamsters were anaesthetized using ketamine (80–100 mg/kg) and xylazine (7–10 mg/kg) and perfused with PBS containing 5 mM EDTA, followed by 4% paraformaldehyde. Tissues of interest were then further fixed according to BSL4 standard operating procedures for a minimum of 7 days in 10% neutral buffered formalin for histopathology or in Karnovksy's fixative (2% paraformaldehyde, 2.5% glutaraldehyde in 0.1 M sodium phosphate) for electron microscopy.

### Histopathology and immunohistochemistry

Histopathology and immunohistochemistry were performed on hamster tissues. After fixation for 7 days in 10% neutral-buffered formalin and embedding in paraffin, tissue sections were stained with hematoxylin and eosin (H&E) staining and an immunohistochemical method using a rabbit polyclonal antiserum against the Nipah virus nucleoprotein (N)^25^(1:5000; kindly provided by L. Wang, CSIRO Livestock Industries, Australian Animal Health Laboratory, Australia) as a primary antibody for detection of Nipah virus antigen. For the histopathological analysis of the nasal turbinates whole hamster skulls were used. The skulls were decalcified using a 20% EDTA solution in sucrose (Newcomer Supply) after removal from BSL4 and allowed to sit at room temperature for 3 weeks. The 20% EDTA/sucrose solution was changed twice prior to bilateral mid-sagittal gross sectioning the skull.

### Transmission electron microscopy

After fixation for 7 days with Karnovsky's fixative at 4°C, excised tissues were post-fixed for 30 minutes with 0.5% osmium tetroxide/0.8% potassium ferricyanide in 0.1 M sodium cacodylate, 1 hour with 1% tannic acid and overnight with 1% uranyl acetate at 4°C. Specimens were dehydrated with a graded ethanol series with two final exchanges in 100% propylene oxide before infiltration and final embedding in Embed-812 resin. Thin sections were cut with a Leica EM UC6 ultramicrotome (Leica, Vienna, Austria), and stained with 1% uranyl acetate and Reynold's lead citrate prior to viewing at 120 kV on a Tecnai BT Spirit transmission electron microscope (FEI, Eindhoven, The Netherlands). Digital images were acquired with a Hammamatsu XR-100 side mount digital camera system (Advanced Microscopy Techniques, Danvers, MA) and processed using Adobe Photoshop v. CS5 (Adobe Systems Inc, San Jose, CA).

For immune labeling, thin sections from specimens prepared as above were collected on 200 mesh nickel grids and the following steps performed by placing grids (section side down) on 20 µl droplets of solutions unless otherwise specified. Sections were etched for 10 minutes with 4% meta-periodate, then incubated with 3% BSA in 0.1 M Tris buffer to block non-specific interactions. Primary anti-Nipah virus N antibody^25^ was diluted 1:100 in 1%BSA/0.1 M Tris and grids incubated on 10 µl droplets for 1 hour. Grids were washed 3×5 minutes with the blocking buffer, and then incubated on 10 µl droplets of goat anti-rabbit 10 nm colloidal gold (BBI International, Cardiff, UK) diluted 1:100 with 1%BSA/0.1 M Tris buffer for 1 hour. Grids were then washed 2×5 minutes with 1%BSA/0.1 M Tris buffer, and then 3×5 minutes with dH2O prior to staining and imaging as described above.

Histopathology and EM were analysed by a board-certified veterinary pathologist with expertise in ultrastructural pathology.

## Author Contributions

Conceived and designed the experiments: VJM, EdW. Performed the experiments: VJM, JBP, TB, DL, RR, TT, DS, ERF, EdW. Analysed the data: VJM, DS, ERF, HF, EdW. Wrote the paper: VJM, HF, EdW.

## Supplementary Material

Supplementary Information Supplementary Information
