# Supplementary material for: Rapid Nipah virus entry into the central nervous system of hamsters via the olfactory route
Source: Sci Rep. 2012 Oct 15;2:736. doi: 10.1038/srep00736 (PMC3471094; doi:10.1038/srep00736)
Supplement: Supplementary Information [file srep00736-s1.pdf]

Supplementary Information

Rapid Nipah virus entry into the central nervous system of Syrian hamsters via the olfactory route

Vincent J. Munster[1*], Joseph B. Prescott[1], Trenton Bushmaker[1], Dan Long[2], Rebecca Rosenke[2], Tina Thomas[1], Dana Scott[2], Elizabeth R. Fischer[3], Heinz Feldmann[1,4] and Emmie de Wit[1*]

[1]Laboratory of Virology, [2]Rocky Mountain Veterinary Branch and [3]Research Technologies Branch, Microscopy Unit, Division of Intramural Research, National Institute of Allergy and Infectious Diseases, National Institutes of Health, Hamilton, MT, USA

[4]Department of Medical Microbiology, University of Manitoba, Winnipeg, Manitoba, Canada

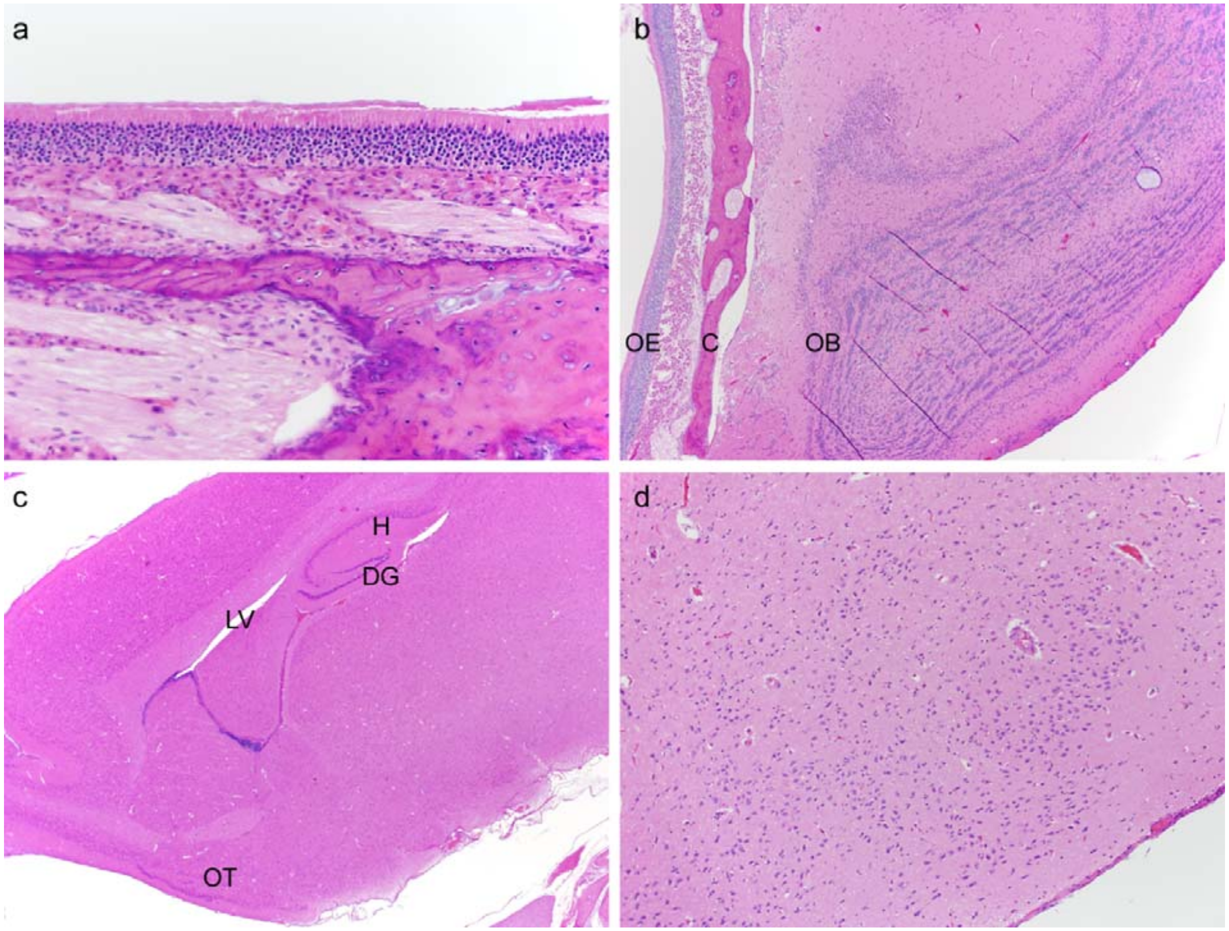

Figure S1. H&E staining of uninfected Syrian hamster control tissues. a) Olfactory epithelium of the nasal turbinates. b) Olfactory epithelium (OE), cribriform plate (C) and olfactory bulb (OB). c) CNS including area of the olfactory tubercle (OT), lateral ventricle (LV), dentate gyrus (DG) and hippocampus (H). d) Close-up of the area of the olfactory tubercle.

Table S1. Histopathology score in hamsters inoculated intranasally with Nipah virus, strain Malaysia.

	Histopathology score																									
	2 dpi				4 dpi				6 dpi				8 dpi				10 dpi			12 dpi			terminal			
Respiratory epithelium[1]	0	0	3	3	4	4	3	3	2	3	2	3	2	3	2	4	4	2	3	1	1	0	3	0	0	1
Olfactory epithelium[1]	2	2	0	2	3	0	4	0	4	4	4	4	0	5	4	5	4	2	4	1	0	0	0	4	4	3
Submucosal glands[1]	0	0	0	0	0	2	3	0	3	3	3	3	0	4	3	3	3	0	3	0	0	0	0	3	3	0
Olfactory bulb	0	0	0	0	0	0	0	0	2	2	2	1	0	1	3	3	3	3	2	3	3	4	3	3	2	3
CNS[2]	0	0	0	0	0	0	0	0	2	2	2	1	2	3	1	3	3	3	2	3	3	4	2	2	3	3
Lungs	0	0	0	3	3	3	3	0	3	4	4	2	2	4	5[3]	1	3	4	0	0	4	3	4	4	4	0

Hamsters were inoculated intranasally with 10^5 TCID50 and sampled at the days post inoculation indicated; four hamsters per dose per time point were sampled except for the 10 and 12 dpi group, when only 3 animals remained; each column represents one animal.

Score: 0: no lesion; 1: minimal change; 2: mild change; 3: moderate change; 4: marked change; 5: severe change

[1] Tissues of the nasal turbinates

[2] Histology score in the CNS excluding the olfactory bulb

[3] Vasculitis present

Table S2. Immunohistochemistry score in hamsters inoculated intranasally with Nipah virus, strain Malaysia.

	Immunohistochemistry score																									
	2 dpi				4 dpi				6 dpi				8 dpi				10 dpi			12 dpi			terminal			
Respiratory epithelium[1]	0	0	0	0	0	1	2	1	0	0	0	2	0	1	0	1	1	0	0	0	0	0	0	0	0	0
Olfactory epithelium[1]	1	2	1	0	1	1	4	1	2	2	2	0	0	0	1	3	0	1	2	0	0	1	0	0	0	0
Submucosal glands[1]	0	0	0	0	2	1	1	2	1	1	1	1	0	1	1	1	0	0	1	0	0	0	0	1	0	0
Olfactory bulb	0	0	0	0	0	0	1	0	0	0	0	0	0	0	4	2	4	-	0	-	0	3	4	-	0	3
CNS[2]	0	0	0	0	0	0	0	0	0	0	1	0	2	0	0	2	0	-	0	0	2	2	1	1	0	0
Lungs	0	0	1	2	2	2	2	0	-	-	-	-	0	0	1	0	-	-	-	-	-	-	-	-	-	-

Hamsters were inoculated intranasally with 10^5 TCID50 and sampled at the days post inoculation indicated; four hamsters per dose per time point were sampled except for the 10 and 12 dpi group, when only 3 animals remained; each column represents one animal.

Score: 0: no immunopositivity; 1: 1 to 25% of tissue immunopositive; 2: 26 to 50% of tissue immunopositive; 3: 51 to 75% of tissue immunopositive; 4: 76 to 100% of tissue immunopositive; -: not examined

[1] Tissues of the nasal turbinates

[2] Histology score in the CNS excluding the olfactory bulb
